# CKD classification based on estimated GFR over three years and subsequent cardiac and mortality outcomes: a cohort study

**DOI:** 10.1186/1471-2369-10-26

**Published:** 2009-09-17

**Authors:** Daniel E Weiner, Maria Krassilnikova, Hocine Tighiouart, Deeb N Salem, Andrew S Levey, Mark J Sarnak

**Affiliations:** 1 Division of Nephrology, Tufts Medical Center, 800 Washington Street, Boston, MA 02111, USA; 2 Department of Medicine Tufts Medical Center 800 Washington Street, Boston, MA 02111, USA

## Abstract

**Background:**

It is unknown whether defining chronic kidney disease (CKD) based on one versus two estimated glomerular filtration rate (eGFR) assessments changes the prognostic importance of reduced eGFR in a community-based population.

**Methods:**

Participants in the Atherosclerosis Risk in Communities Study and the Cardiovascular Health Study were classified into 4 groups based on two eGFR assessments separated by 35.3 ± 2.5 months: sustained eGFR < 60 mL/min per 1.73 m^2 ^(1 mL/sec per 1.73 m^2^); eGFR increase (change from below to above 60); eGFR decline (change from above to below 60); and eGFR persistently ≥60. Outcomes assessed in stratified multivariable Cox models included cardiac events and a composite of cardiac events, stroke, and mortality.

**Results:**

There were 891 (4.9%) participants with sustained eGFR < 60, 278 (1.5%) with eGFR increase, 972 (5.4%) with eGFR decline, and 15,925 (88.2%) with sustained eGFR > 60. Participants with eGFR sustained < 60 were at highest risk of cardiac and composite events [HR = 1.38 (1.15, 1.65) and 1.58 (1.41, 1.77)], respectively, followed by eGFR decline [HR = 1.20 (1.00, 1.45) and 1.32 (1.17, 1.49)]. Individuals with eGFR increase trended toward increased cardiac risk [HR = 1.25 (0.88, 1.77)] and did not significantly differ from eGFR decline for any outcome. Results were similar when estimating GFR with the CKD-EPI equation.

**Conclusion:**

Individuals with persistently reduced eGFR are at highest risk of cardiovascular outcomes and mortality, while individuals with an eGFR < 60 mL/min per 1.73 m^2 ^at any time are at intermediate risk. Use of even a single measurement of eGFR to classify CKD in a community population appears to have prognostic value.

## Background

Chronic kidney disease (CKD) is defined by either evidence of kidney damage, including microalbuminuria, or by glomerular filtration rate (GFR) below 60 mL/min per 1.73 m^2 ^(1 mL/sec per 1.73 m^2^), with the requirement that these persist for at least 3 months [1]. Although two reports from clinical databases present sensitivity analyses accounting for repeated measures of creatinine assessed in subsets of their study populations requiring more frequent clinical evaluations [2,3] and a report from a third clinical database examines the affect of classifying CKD using creatinine measured at varying time intervals as dictated by clinical practice [4], most community-based cohorts linking CKD with subsequent cardiovascular disease and mortality use a single measurement of baseline serum creatinine to define CKD [5-9]. Critically, reliance on a single measurement or non-systematic ascertainment of serum creatinine to define disease prevalence may result in misclassification. For example, although a single creatinine measurement was used to estimate the US prevalence of CKD in the National Health and Nutrition Evaluation Surveys (NHANES) [10,11], in the subset of NHANES III where serum creatinine was measured twice over a median of 17 days, creatinine values differed by 0.2% ± 9.7% [10]. Similarly, if microalbuminuria on a single urine specimen rather than persistent microalbuminuria defined stage 1 and 2 CKD in NHANES III, as many as 6.4 million additional individuals in the US could be classified as having CKD [12].

While evidence suggests that a rapid decrement in kidney function is associated with a significant increased risk of cardiovascular and all-cause mortality in older adults [13], the chronicity of stage 3 CKD classification and the prognostic significance of this chronicity over a three-year interval has not been carefully studied in a community-based cohort. In epidemiologic cohorts, it remains unknown whether classification of CKD based on one versus two measurements results in a different set of individuals, and whether classification based on two measurements carries different prognostic importance. The utility in defining a disease state is that, once identified, those individuals with that condition can be 1) designated as higher risk for sequelae of that condition and 2) treated for this risk (1). Therefore, regardless of misclassification, if a one-time measure of estimated GFR (eGFR) consistent with CKD identifies individuals at increased risk of the common sequelae of CKD, specifically cardiovascular disease and mortality, its importance remains.

Accordingly, in the current study, we evaluate the stability of CKD classification, based on National Kidney Foundation Kidney Disease Outcomes Quality Improvement (KDOQI) guidelines [1], using two creatinine measurements over three years in individuals from 2 large community-based cohorts, the Atherosclerosis Risk in Communities (ARIC) Study and the Cardiovascular Health Study (CHS). We then assess the cardiovascular disease and mortality risk associated with four kidney function groups based on eGFR change over time: sustained eGFR < 60 mL/min per 1.73 m^2^, with eGFR that changes from below to above 60 mL/min per 1.73 m^2^, with eGFR that drops below 60 mL/min per 1.73 m^2 ^after having been above 60 mL/min per 1.73 m^2^, and with eGFR persistently ≥60 mL/min per 1.73 m^2^.

## Methods

### Study Population

Individual patient data were pooled from 2 community-based, longitudinal studies, ARIC and CHS, available as de-identified data from the US National Institutes of Health. ARIC recruited 15,792 subjects, ages 45 to 64 years, between 1987 and 1989. CHS included 5,201 subjects, 65 years and older, randomly selected from Medicare eligibility files during 1989 and 1990. In both studies, follow-up occurred at 3-4 year intervals; data from the initial and the first follow-up visits are used in this analysis. An additional 687 African American participants were recruited in CHS from 1992-1993; they were not included here due to limited follow-up. Further details of these studies are described elsewhere [14,15].

### Creatinine Calibration

In ARIC, serum creatinine was assessed in 15,582 (99%) subjects at their initial visit, while in CHS it was assessed in 5,716 (97%) subjects. We indirectly calibrated mean individual first visit creatinine values from ARIC and CHS to mean NHANES III for a given age, race and sex, following a fixed offset of -0.23 mg/dL (20 μmol/L) to calibrate to Cleveland Clinic values, resulting in adjustments of -0.24 mg/dL (21 μmol/L) in first visit ARIC values and -0.11 mg/dL (10 μmol/L) in first visit CHS values [16].

Because informative censoring from death and dropout results in a non population-based sample, second visit measurements cannot be calibrated to NHANES values in the same manner. In ARIC, second visit serum creatinine values were adjusted by -0.24 mg/dL (21 μmol/L) according to published data [17]. In CHS, the first visit for the African American cohort and the second visit for the original cohort were concurrent. As creatinine calibration is performed to account for assay differences and there should not be a difference in calibration factor by race, we indirectly calibrated the African American cohort to African American participants in NHANES III as described above. This calibration model showed that serum creatinine values were 0.04 mg/dL (3.5 μmol/L) greater in the CHS African-American cohort than NHANES III; accordingly, we subtracted this value from second visit measurements in the CHS cohort. Estimated GFR was calculated with the 4-variable Modification of Diet in Renal Disease (MDRD) Study equation [18].

Using these two eGFR values, participants were then classified into 4 groups: 1) eGFR < 60 mL/min per 1.73 m^2 ^(eGFR < 60 mL/min per 1.73 m^2 ^at both visits); 2) eGFR≥ 60 mL/min per 1.73 m^2 ^(≥60 mL/min per 1.73 m^2 ^at both visits); 3) eGFR increase ( < 60 mL/min per 1.73 m^2 ^at first visit and ≥60 mL/min per 1.73 m^2 ^at second visit); and 4) eGFR decline (≥60 mL/min per 1.73 m^2 ^at first visit and < 60 mL/min per 1.73 m^2 ^at second visit).

### Baseline Covariates

Other baseline variables included demographics (age, sex, race, education status), lifestyle characteristics (smoking, alcohol intake), glycemic and antihypertensive medication use, past medical history (diabetes, hypertension and cardiovascular disease), examination findings (systolic and diastolic blood pressure, waist-to-hip ratio (WHR), electrocardiogram results); and blood laboratory variables (total cholesterol, high density lipoprotein (HDL) cholesterol, albumin, glucose). Second visit data were used in multivariable models for all variables except albumin where 1^st ^visit data were used.

Race was defined as white or African American. Education level was dichotomized by high school graduation status. Cigarette smoking was stratified as never, former or current, and alcohol use was dichotomized by current use. Diabetes was defined by self-reported history, use of oral hypoglycemic agents or insulin, or fasting glucose ≥126 mg/dL (7 mmol/L). Hypertension was defined by systolic blood pressure ≥140 mm Hg, diastolic ≥90 mm Hg or use of antihypertensive medications. WHR was calculated by dividing waist circumference by hip circumference. Left ventricular hypertrophy (LVH) was defined by electrocardiographic criteria [19]. History of cardiovascular disease was defined by prior recognized or silent myocardial infarction, angina based on the Rose questionnaire, stroke, transient ischemic attack, intermittent claudication, and/or prior coronary angioplasty or bypass procedures.

### Study Outcomes

The primary outcome was a composite of cardiac events (myocardial infarction, coronary revascularization or fatal coronary disease), stroke, or all-cause mortality. Secondary outcomes included individual components of the primary composite outcome. ARIC only identifies time to the first cardiac and stroke event and does not provide data on subsequent events. Therefore, those participants who had a cardiac event (n = 178) or a stroke (n = 62) between their first and second visits were defined as having a history of CVD but were excluded from analyses examining future cardiac or stroke outcomes; these 235 individuals (5 had both a cardiac event and stroke) were only included in analyses examining mortality.

### Study Sample

**Figure 1:**
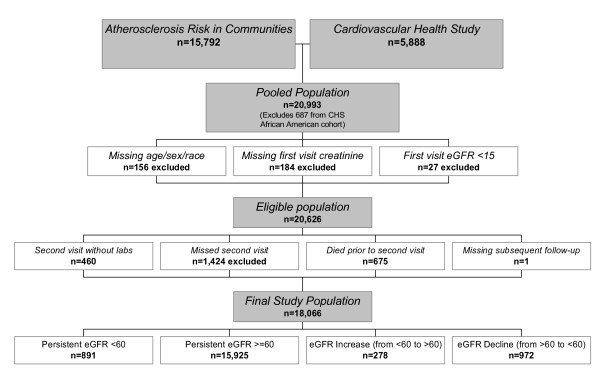
**Derivation of the study population**. eGFR, estimated glomerular filtration rate in mL/minute per 1.73m^2^.

From a pooled sample of 21,680 individuals, we excluded the African American cohort from CHS enrolled at the time of the second visit (n = 687). Of the remaining 20,993 participants, we excluded 156 who were missing age, sex or race data, 184 missing first visit creatinine, and 27 with first visit eGFR < 15 mL/min per 1.73 m^2 ^to avoid inclusion of dialysis patients, yielding 20,626 eligible participants. Of these, 2,560 (12.4%) were missing eGFR at their second visit, with reasons including death prior to the expected follow-up time (n = 675), no reported laboratory results (n = 460), no data after the second visit (n = 1), and no second visit (n = 1,424; 1,078 from ARIC and 346 from CHS), yielding a final study population of 18,066 individuals used in univariate analyses (Figure 1). There were 17,698 participants with no missing covariates used in multivariable analyses.

### Statistical Analysis

Second visit characteristics were compared with analysis of variance for continuous variables and chi-square tests for categorical variables. Proportions of individuals falling into each kidney function classification were calculated and stability of classifications was defined by remaining above or below 60 mL/min per 1.73m^2^. As a sensitivity analysis, among participants missing a second GFR estimate the minimal and maximal variation in stability of classification of sustained eGFR < 60 was estimated by assuming that a) eGFR remained below 60 mL/min per 1.73m^2 ^from the first to the second visit and b) eGFR changed groups from below to above 60 mL/min per 1.73m^2^.

Event rates were calculated and Kaplan-Meier survival analysis was used to estimate the nonparametric survival distribution among study participants by eGFR group beginning at the time of the second GFR estimate. Cox proportional hazards regression utilized the SAS procedure 'TPHREG' with a class statement for eGFR group to examine differences in study outcomes among the respective comparison groups while adjusting for covariates. All models *a priori *included the following: age, sex, race, education, study of origin; smoking and drinking status; diabetes, hypertension, and cardiovascular disease history; systolic blood pressure, WHR, and LVH; and non-HDL cholesterol and albumin. In additional analyses evaluating models that revealed no significant differences in hazards for study outcomes between individuals with eGFR decline and eGFR increase, these two groups were combined and analyses repeated with a 3-level exposure term that also included sustained eGFR < 60 and sustained eGFR ≥60 mL/min per 1.73m^2^. The proportional hazards assumption was checked by testing the significance of the correlation coefficient between survival time for the composite outcome and the scaled Schoenfeld residuals using a chi-square statistic with a two-sided p-value and was met for all covariates.

Because prior research has found less consistent relationships between individuals with eGFR between 50 and 59 and adverse outcomes [3], we performed sensitivity analyses assessing study outcomes in individuals with eGFR sustained ≥60 mL/min per 1.73m^2^, individuals with eGFR sustained between 50 and 59 mL/min per 1.73m^2^, and individuals with eGFR sustained below 50 mL/min per 1.73m^2^. We also tested the effect of including the initial eGFR in multivariable models. Lastly, we performed a second series of analyses that duplicated the primary analyses but utilized eGFR calculated with the CKD-EPI estimating equation rather than the 4-variable MDRD equation after indirect calibration of serum creatinine from a non-IDMS to an IDMS standard [20].

All analyses were performed with SAS version 9.1. The Institutional Review Board at Tufts Medical Center approved this research.

## Results

### Study Participants

**Figure 2:**
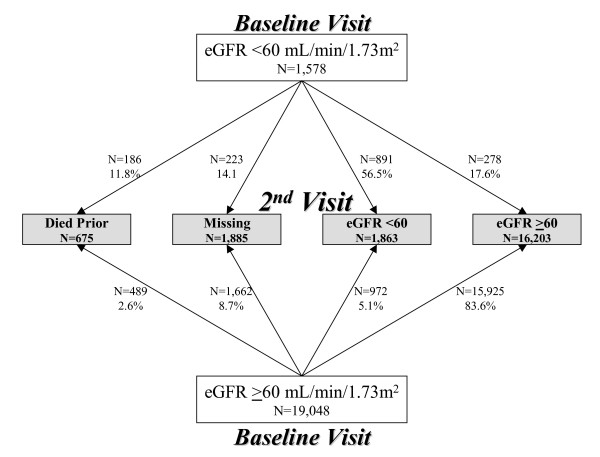
**Transitions from the first to second study visit by baseline eGFR group**. 'Missing' refers to participants either without second visit labs or without a second visit altogether.

There were 20,626 participants with first visit eGFR ≥15 mL/min per 1.73m^2^, 18,066 of whom had two GFR estimates (Figure 2). First visit eGFR was < 60 mL/min per 1.73m^2 ^in 409 (16.0%) of the 2,560 individuals missing a second visit eGFR compared to 1,169 (6.5%) of the 18,066 individuals with a second visit eGFR (p < 0.0001). Among 1,578 participants with first visit eGFR below 60 mL/min per 1.73m^2^, 186 (11.8%) died prior to the second visit, while, among 19,048 participants with first visit eGFR above 60 mL/min per 1.73m^2^, 489 (2.6%) died prior to the second visit (p < 0.0001). Participants missing second visit data had a higher prevalence of cardiovascular disease risk factors than those with second visit data. Among participants who died prior to the second visit, mean age was 67.8 years and 41.7% had a history of CVD, 28.2% had diabetes and 73.2% had hypertension. Among those alive but missing their second visit, mean age was 62.1 years and 21.9% had a history of CVD, 17.0% had diabetes and 56.2% had hypertension. This compares to first visit mean age 58.2 ± 9.5 years, CVD prevalence of 15.3%, diabetes prevalence 10.6% and hypertension prevalence of 43.3% among participants who subsequently had a second study visit (p < 0.0001 for all comparisons).

### Chronicity of eGFR Classification

**Figure 3:**
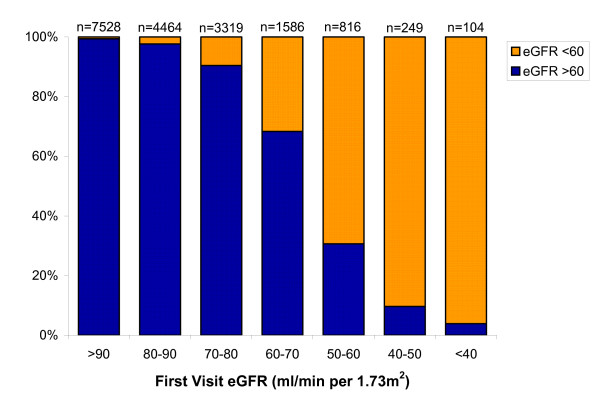
**Proportion of individuals with eGFR < 60 mL/min per 1.73m^2 ^or ≥ 60 mL/min per 1.73m^2 ^at their second study visit stratified by the initial eGFR**. GFR is estimated using the 4-variable MDRD equation. The x-axis refers to the eGFR at the first study visit while the y-axis identifies the proportion of individuals with that initial eGFR who had a second visit eGFR above and below 60 mL/min per 1.73m^2^. 'n' refers to the number of participants in each eGFR group at the first study visit.

Among those with longitudinal data, there were 1,169 participants with first visit eGFR < 60 mL/min per 1.73m^2^. Of these individuals, 891 (76.2%) had eGFR persistently below this level at follow-up while 278 (23.8%) had eGFR that rose above 60 mL/min per 1.73m^2 ^at follow-up (Figure 3). As a sensitivity analysis, if none of the 409 individuals with baseline eGFR < 60 mL/min per 1.73m^2 ^and missing subsequent eGFR had persistent eGFR < 60 mL/min per 1.73m^2^, the stability of stage 3 CKD identification based on a single assessment could be as low as 56.5% (891/1,578) while, if all 409 individuals had persistently low eGFR, persistence could be as high as 82.4% (1,300/1,578).

Numbers of individuals with persistently reduced eGFR, with eGFR below 60 mL/min per 1.73m^2 ^at the first visit and ≥60 mL/min per 1.73m^2 ^at the second visit (eGFR increase), with eGFR ≥60 mL/min per 1.73m^2 ^at the first visit and < 60 mL/min per 1.73m^2 ^at the second visit (eGFR decline), and with both measurements ≥60 mL/min per 1.73m^2 ^are shown in Figure 2. Mean time between measurements was 35.3 ± 2.5 months and was similar across groups (p = 0.8). Participants with sustained eGFR < 60 were older, had higher systolic blood pressure, and more likely to be white and diabetic than those with eGFR persistently > 60 mL/min per 1.73m^2^. Those who experienced eGFR decline had significantly lower baseline eGFR than those eGFR persistently > 60 mL/min per 1.73m^2^; additionally, those with GFR decline were significantly older, more likely to be female, more frequently had a history of diabetes, cardiovascular disease, and hypertension, and had higher systolic blood pressure. Individuals with an increase in eGFR were similar to those who experienced an eGFR decline (Table 1).

**Table 1:** Demographic and Clinical Characteristics at the Time of the Second Study Visit

	eGFR < 60 (n = 891, 4.9%)	eGFR ≥ 60 (n = 15,925, 88.1%)	eGFR Increase (n = 278, 1.5%)	eGFR Decline (n = 972, 5.4%)	Total (n = 18,066)
Demographics					
Age	73.4 ± 9.1	59.9 ± 8.7	65.5 ± 9.7	69.0 ± 11.0	61.1 ± 9.5
Female	54.9	55.3	64.0	59.5	55.7*
African American	7.3	21.3	9.0	10.9	19.8
High School Graduate	73.7	78.1	74.1	74.7	77.7^†^
ARIC	24.7	83.5	53.2	47.2	78.2
Visit Interval, months	35.3 ± 1.6	35.3 ± 2.6	35.3 ± 2.0	35.4 ± 2.1	35.3 ± 2.5^§^
					
Medical History					
Diabetes	18.4	14.7	20.9	18.6	15.2
Hypertension	77.2	43.1	61.2	68.1	46.4
CVD	35.0	14.8	27.0	27.0	16.6
Current Smoker	9.8	20.8	15.6	10.3	19.6
Former Smoker	49.0	38.7	48.2	43.9	39.7
Current Alcohol Use	43.6	55.5	46.4	47.9	54.3
					
Physical Findings					
Systolic BP	136 ± 24	124 ± 20	128 ± 20	131 ± 23	125 ± 20
Diastolic BP	71 ± 12	72 ± 10	71 ± 10	71 ± 12	72 ± 11^‡^
LVH	6.3	2.5	3.3	5.9	2.9
Body Mass Index	26.8 ± 4.6	27.6 ± 5.1	27.6 ± 5.0	27.5 ± 4.8	27.5 ± 5.1^†^
Waist to Hip Ratio	0.95 ± 0.07	0.93 ± 0.08	0.94 ± 0.07	0.94 ± 0.08	0.93 ± 0.08
					
Laboratory Results					
V1 Creatinine	1.3 ± 0.3	0.8 ± 0.2	1.2 ± 0.2	1.0 ± 0.2	0.9 ± 0.2
V1 eGFR	50.8 ± 7.7	93.7 ± 20.0	55.9 ± 4.6	72.2 ± 11.0	89.8 ± 22.0
V2 Creatinine	1.5 ± 0.8	0.9 ± 0.2	1.0 ± 0.2	1.3 ± 0.3	0.9 ± 0.3
V2 eGFR	47.4 ± 9.6	86.1 ± 17.1	68.2 ± 8.5	53.8 ± 6.6	82.1 ± 19.6
Hematocrit	40.2 ± 4.3	40.8 ± 3.7	40.8 ± 3.8	40.4 ± 4.1	40.8 ± 3.7
Total Cholesterol	210.2 ± 42.4	209.2 ± 38.9	210.8 ± 41.1	214.7 ± 43.3	209.6 ± 39.4
HDL Cholesterol	49.2 ± 14.6	50.3 ± 16.4	49.6 ± 16.6	49.4 ± 15.4	50.1 ± 16.3^§^
Albumin	4.0 ± 0.3	3.9 ± 0.3	4.0 ± 0.3	3.9 ± 0.3	3.9 ± 0.3

CVD, cardiovascular disease; BP, blood pressure; V1, first visit; V2, second visit (which serves as the baseline visit); LVH, left ventricular hypertrophy; eGFR, estimated glomerular filtration rate. Continuous variables are mean ± standard deviation and categorical variables are %. P-values are for differences among groups. Age is in years, blood pressures in mm Hg, body mass index in kg/m^2^, waist to hip in cm/cm, creatinine and cholesterols in mg/dL, GFR in mL/min per 1.73m^2^, hematocrit in %, and albumin in g/dL. Albumin was not present at the 2^nd ^visit in both studies; therefore values represent the 1^st ^study visit.

To convert creatinine to μmol/L, multiply by 884; to convert eGFR to mL/sec per 1.73 m2, multiply by 0.01667; to convert hemoglobin and albumin to g/L, multiply by 10; to covert cholesterols to mmol/L, multiply by 0.02586.

All p-values < 0.0001 except: *, < 0.01; ^†^, < 0.001l; ^‡^, < 0.05; ^§^, non-significant

### Cardiovascular and Mortality Outcomes

**Figure 4:**
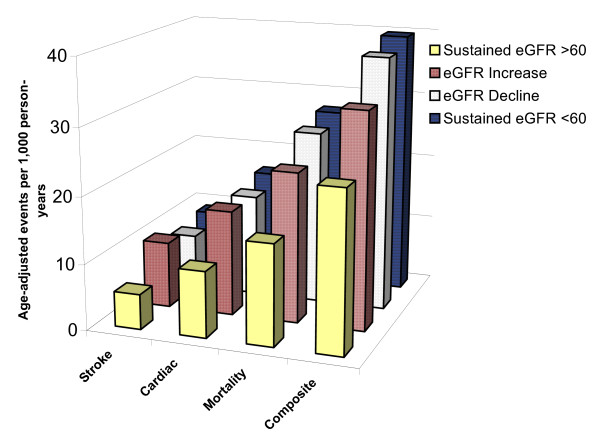
**Age-adjusted event rates per 1,000 person-years, calculated using the SAS^® ^PROC GENMOD with the Poisson distribution option and adjusting for varying follow-up time of individual participants**.

**Figure 5:**
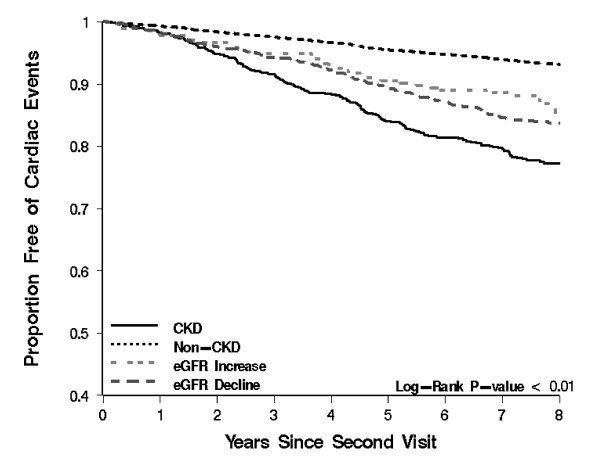
**Kaplan-Meier curves presenting the relationship between kidney function groups and Cardiac outcomes**.

**Figure 6:**
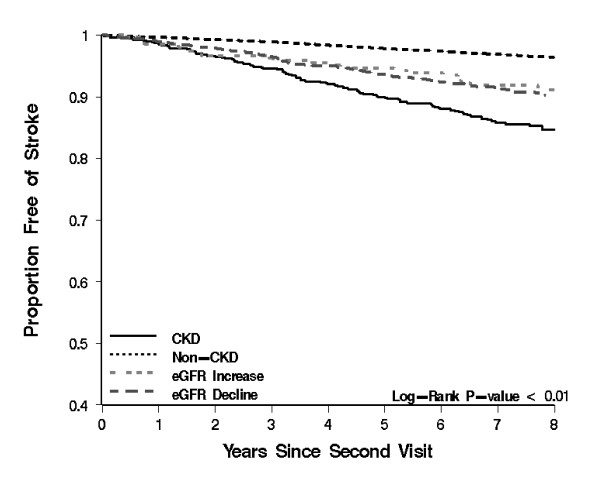
**Kaplan-Meier curves presenting the relationship between kidney function groups and Stroke outcomes**.

**Figure 7:**
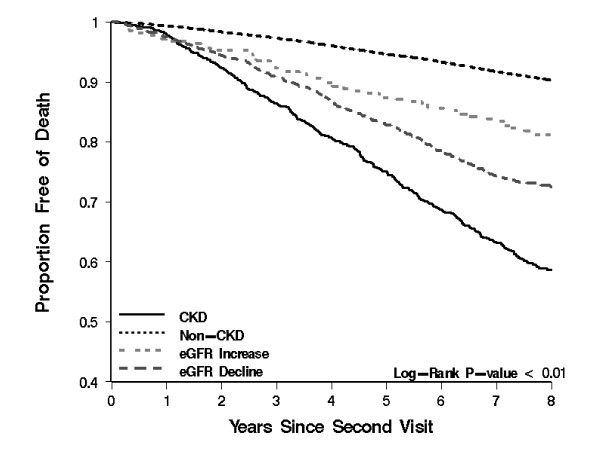
**Kaplan-Meier curves presenting the relationship between kidney function groups and Mortality outcomes**.

**Figure 8:**
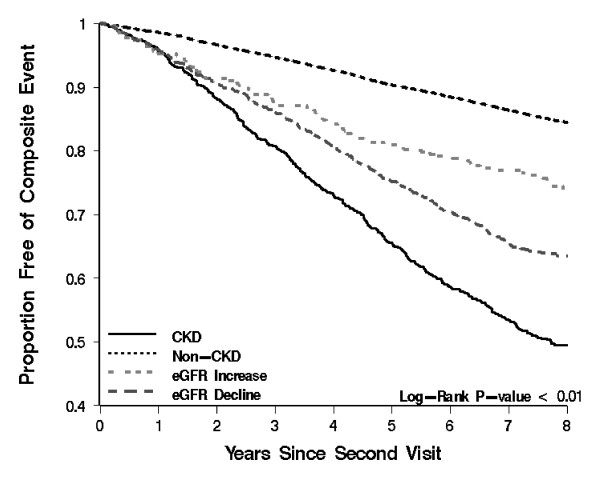
**Kaplan-Meier curves presenting the relationship between kidney function groups and Composite outcomes**.

Event rates for all outcomes were highest among those with sustained eGFR < 60, followed by those with eGFR decline and eGFR increase; event rates were lowest for those eGFR persistently > 60 mL/min per 1.73m^2 ^(Figure 4). Results of univariate analyses are presented in Table 2 and Figure 5, 6, 7 and 8 and reveal a graded risk of adverse outcomes based on kidney function group. Individuals with sustained eGFR < 60 mL/min per 1.73m^2 ^were at greatest risk.

**Table 2:** Adverse Events and Unadjusted Hazards for Cardiac, Stroke, Mortality and Composite Events Based on Kidney Function Classification

		eGFR≥60	eGFR Increase	eGFR Decline	eGFR < 60
**Cardiac**	*events/at risk; %*	1,088/15,767; 6.9%	36/276; 13.0%	143/961; 14.9%	175/884; 19.8%
	HR (CI)	Reference	2.10 (1.50, 2.92)	2.48 (2.08, 2.95)	3.66 (3.12, 4.30)
	
**Stroke**	*events/at risk; %*	568/15,870; 3.6%	23/277; 8.3%	81/970; 8.4%	114/887; 12.9%
	HR (CI)	Reference	2.60 (1.71, 3.94)	2.73 (2.16, 3.44)	4.68 (3.82, 5.72)
	
**Mortality**	*events/at risk; %*	1,612/15,925; 10.1%	53/278; 19.1%	269/972; 27.7%	367/891; 41.2%
	HR (CI)	Reference	2.10 (1.60, 2.76)	3.16 (2.78, 3.60)	5.27 (4.70, 5.90)
	
**Composite**	*events/at risk; %*	2,584/15,925; 16.2%	72/278; 25.9%	356/972; 36.6%	451/891; 50.6%
	HR (CI)	Reference	1.81 (1.43, 2.28)	2.68 (2.40, 2.99)	4.17 (3.77, 4.61)

HR, Hazard Ratio; CI, 95% confidence interval

Lower 'at risk' numbers for cardiac and stroke outcomes reflect 178 and 62 individuals, respectively, who had a cardiac event and a stroke between visits 1 and 2.

Difference between eGFR increase and decline non-significant for cardiac (p = 0.37) and stroke outcomes (p = 0.84) and significant for mortality (p = 0.006) and composite outcomes (0.002).

In multivariable analyses, individuals with sustained eGFR < 60 mL/min per 1.73m^2 ^were at highest risk for all outcomes followed by those with eGFR decline (Table 3). Individuals with eGFR increase trended to increased cardiac and stroke risk in multivariable models. There was no significant difference in the hazard ratios associated with eGFR decline and eGFR increase in fully adjusted models for any of the four study outcomes, although this may have been limited by power. When participants classified as eGFR increase or eGFR decline were considered as a single group in order to assess the association of having an eGFR below 60 mL/min per 1.73m^2 ^at any point with outcomes, this combined population was at significantly increased risk of all study outcomes compared to those with eGFR persistently ≥60 mL/min per 1.73m^2 ^[Hazard Ratio (HR) = 1.21 (1.02,1.44) for cardiac outcomes; HR = 1.30 (1.04,1.63) for stroke; HR = 1.38 (1.21,1.58) for death; and HR = 1.28 (1.14,1.43) for composite outcomes in fully adjusted models]. Inclusion of baseline eGFR in the multivariable models did not substantially affect the hazard ratios associated with the eGFR groups (Table 4).

**Table 3:** Results of Multivariable Models for Time to Cardiac, Stroke, Mortality and Composite Events [Hazard Ratio (95% Confidence Interval)].

	Cardiac	Stroke	Mortality	Composite
eGFR < 60	1.38 (1.15, 1.65)	1.49 (1.18, 1.87)	1.83 (1.61, 2.08)	1.58 (1.41, 1.77)
eGFR Decline	1.20 (1.00, 1.45)	1.51 (0.97, 2.35)	1.42 (1.24, 1.64)	1.32 (1.17, 1.49)
eGFR Increase	1.25 (0.88, 1.77)	1.25 (0.97, 1.61)	1.21 (0.91, 1.62)	1.09 (0.85, 1.40)
eGFR ≥60	Reference	Reference	Reference	Reference

Models are adjusted for age, sex, race, study, education, diabetes, history of cardiovascular disease, history of hypertension, alcohol use, smoking status, systolic blood pressure, waist-to-hip ratio, left ventricular hypertrophy, non-HDL cholesterol, and albumin.

Differences between eGFR increase and decline groups non-significant for cardiac (p = 0.86), stroke (p = 0.45), mortality (p = 0.31), and composite (0.16) outcomes.

**Table 4:** Results of sensitivity analyses including baseline eGFR calculated using the 4-variable MDRD equation in multivariable models

	Cardiac	Stroke	Mortality	Composite
eGFR < 60	1.26 (1.01, 1.56)	1.60 (1.20, 2.13)	2.16 (1.84, 2.55)	1.73 (1.51, 1.99)
eGFR Decline	1.15 (0.95, 1.40)	1.29 (1.00, 1.68)	1.54 (1.33, 1.79)	1.38 (1.22, 1.57)
eGFR Increase	1.15 (0.79, 1.66)	1.62 (1.01, 2.58)	1.42 (1.05, 1.93)	1.19 (0.92, 1.55)
eGFR ≥60	Reference	Reference	Reference	Reference
Visit 1 eGFR	0.98 (0.94, 1.01)	1.02 (0.98, 1.06)	1.05 (1.02, 1.07)	1.03 (1.00, 1.05)

Models adjusted for all variables listed in table 3 as well as visit 1 eGFR.

### Mild Sustained Decreased eGFR and Outcomes

There were 338 individuals with eGFR that remained between 50 and 59 mL/min per 1.73m^2 ^at both the first and second study visit and 250 individuals with eGFR < 50 mL/min per 1.73m^2 ^at both study visits. In fully adjusted models, compared to those with eGFR sustained ≥60 mL/min per 1.73m^2^, individuals with a mild sustained decrease in eGFR were at significantly increased risk of composite events and all-cause mortality [HR = 1.28 (1.06,1.55) and 1.45 (1.17,1.72), respectively] and trended to increased risk of cardiac and stroke events [HR = 1.27 (0.96,1.69) and 1.33 (0.92,1.92), respectively]. Of note, there were only 57 cardiac events and 35 strokes in these individuals. Individuals with eGFR sustained < 50 mL/min per 1.73m^2 ^were at significantly increased risk of all study outcomes when compared to those with sustained ≥60 mL/min per 1.73m^2 ^(data not shown).

### GFR estimated using the CKD-EPI Equation

**Figure 9:**
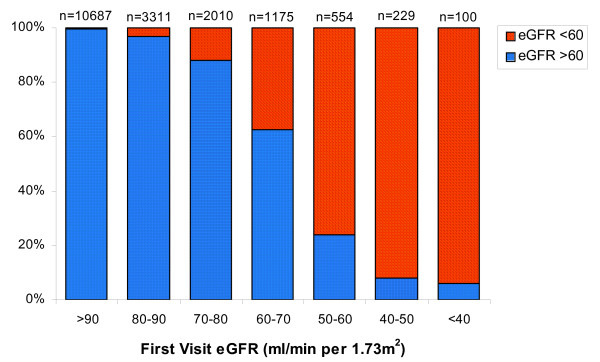
**Proportion of individuals with eGFR < 60 mL/min per 1.73m^2 ^or ≥ 60 mL/min per 1.73 m^2 ^at their second study visit stratified by the initial eGFR**. GFR is estimated using the CKD-EPI equation. The x-axis refers to the eGFR at the first study visit while the y-axis identifies the proportion of individuals with that initial eGFR who had a second visit eGFR above and below 60 mL/min per 1.73m^2^. 'n' refers to the number of participants in each eGFR group at the first study visit.

There was 96.6% agreement in classification between the MDRD and CKD-EPI prediction equations; the notable difference was a decrease in the prevalence of individuals with sustained eGFR below 60 mL/min per 1.73m^2^, as well as decreases in both the eGFR decrease and increase groups, with a corresponding rise in the sustained eGFR ≥60 mL/min per 1.73m^2 ^group (Table 5). The proportion of individuals with eGFR < 60 mL/min per 1.73m^2 ^or ≥60 mL/min per 1.73m^2 ^at their second study visit stratified by the initial eGFR is presented in Figure 9. The relationship between the kidney function groups and study outcomes were similar to those seen when using the MDRD equation (Table 6).

**Table 5:** Cross-tabulation of eGFR strata using the 4 variable MDRD estimating equation and the CKD-EPI estimating equation.

		CKD-EPI				
		**eGFR < 60**	**eGFR ≥ 60**	**eGFR Increase**	**eGFR Decline**	**Total**
		
MDRD	eGFR < 60	710	42	25	114	891
	eGFR ≥60	2	15,900	5	18	15,925
	eGFR Increase	1	150	127	0	278
	eGFR Decline	13	243	0	716	972
		
	Total	726	16,335	157	848	18,066

eGFR, estimated glomerular filtration rate in mL/min per 1.73m^2^. Cross-tabulation is in bold.

**Table 6:** Results of Multivariable Models for Time to Cardiac, Stroke, Mortality and Composite Events [Hazard Ratio (95% Confidence Interval)] with eGFR groups determined using the CKD-EPI estimating equation.

	Cardiac	Stroke	Mortality	Composite
eGFR < 60	1.41 (1.15, 1.65)	1.47 (1.15, 1.86)	1.81 (1.59, 2.08)	1.60 (1.42, 1.80)
eGFR Decline	1.14 (0.94, 1.39)	1.25 (0.97, 1.62)	1.52 (1.32, 1.75)	1.35 (1.19, 1.52)
eGFR Increase	1.50 (1.04, 2.19)	1.57 (0.96, 2.55)	1.34 (0.98, 1.82)	1.30 (0.99, 1.70)
eGFR ≥60	Reference	Reference	Reference	Reference

Models are adjusted for age, sex, race, study, education, diabetes, history of cardiovascular disease, history of hypertension, alcohol use, smoking status, systolic blood pressure, waist-to-hip ratio, left ventricular hypertrophy, non-HDL cholesterol, and albumin.

## Discussion

The current study demonstrates that individuals with persistent reduction in eGFR are at highest risk of cardiovascular outcomes and mortality, while classifying individuals with an eGFR < 60 mL/min per 1.73m^2 ^at any time as being at intermediate risk of cardiovascular events and mortality. We also demonstrate that there is a moderate lack of chronicity in classification of CKD based on a single assessment in this longitudinal cohort, with 23.8% of individuals with baseline eGFR below 60 mL/min per 1.73m^2 ^who were able to attend a follow-up 3 years later having eGFR ≥60 mL/min per 1.73m^2 ^at that time.

There are several findings in this study that are important. First, we confirmed the risk relationship of reduced eGFR, estimated both with the MDRD and CKD-EPI estimating equations, with cardiac, stroke and death events that has been described previously [2,8]. Second, we demonstrated that there is some inconsistency when defining CKD with a single measurement, with potential reclassification occurring in 24% of individuals who would be defined as having stage 3 CKD based on the first creatinine measurement. Not surprisingly, these individuals had GFR estimates at baseline that were close to the 60 mL/min per 1.73m^2 ^threshold used by clinical guidelines to define stage 3 CKD and were disproportionately women when compared to those with eGFR sustained < 60 mL/min per 1.73m^2^. Third, despite potential reasons for misclassification, individuals with an eGFR below 60 mL/min per 1.73m^2 ^at either visit were at substantially increased risk of adverse outcomes in univariate analyses, those with an eGFR decline were at increased risk in multivariable analysis, and even those with an eGFR increase showed trends toward increased risk in multivariable analyses. Fourth, we demonstrated that categorization based on the most recent GFR estimate likely is a better predictor of outcomes, as individuals with eGFR decline, in comparison to those with eGFR increase, were at higher risk of death and composite outcomes in univariate analysis and showed trends toward higher risk of composite outcomes in multivariable analysis. Similar relationships have been demonstrated with the utility of albuminuria for predicting incident kidney failure [21]. Fifth, we were able to demonstrate that individuals with a mild sustained reduction in eGFR (50-59 mL/min per 1.73m^2^) were at increased risk of adverse outcomes when compared to individuals with eGFR sustained > 60 mL/min per 1.73m^2^.

By exploring multiple GFR estimates in a well-defined, widely generalizable population with systematic ascertainment of comorbid conditions, laboratory variables and clinical outcomes, our study adds to previous findings that were predominantly derived from databases of repeated GFR estimates in populations receiving clinical care in large healthcare systems. Recently, Eriksen and Ingebretsen analyzed creatinine measurements obtained as a part of routine clinical activity in North Norway and noted that, as the interval between GFR estimates rose from 3 month to 12 months, the proportion of individuals with baseline eGFR between 30 and 60 mL/min per 1.73m^2 ^who subsequently experienced an improvement in eGFR to ≥60 mL/min per 1.73m^2 ^dropped from 4.8% to 2.3% [4]. Our study also adds substantially to the findings of Go and colleagues and O'Hare and colleagues [2,3]. O'Hare and colleagues note that, among the 30% of their population with multiple serum creatinine measures within 6 months of the baseline assessment, 81% remained within the same eGFR category while 8% had moved to a higher eGFR category and 11% to a lower eGFR category. Importantly, O'Hare and colleagues also noted that individuals with stable eGFR between 50 and 60 mL/min per 1.73m^2 ^did not have an increased risk of mortality when compared to individuals with eGFR > 60 mL/min per 1.73m^2^. Our findings differ in this regard, perhaps reflecting the different nature of clinical and epidemiologic cohorts as well as the different duration between creatinine measurements.

Using a single baseline measure of serum creatinine to estimate GFR for purposes of determining CKD stage and the risk associated with reduced kidney function offers reasonable accuracy. Although, given the number of individuals who did not have a second GFR estimate, it is possible that eGFR may be persistently low as little as 56.5% of the time, it is more likely that informative censoring occurred as individuals without a second eGFR were more likely to have an adverse event and reduced eGFR has been shown in multiple studies to be associated with poor outcomes [2,8,22]. This suggests that a single eGFR measurement can identify stage 3 or higher CKD in a population-based sample approximately 75-80% of the time. This is important information for interpreting prior literature that has relied on only a single baseline creatinine assessment to estimate kidney function and CKD [11].

One weakness of this study is our dependence on indirect calibration of serum creatinine for determination of longitudinal changes in eGFR. While we used the best available methodology for calibration, our calibration corrections remain dependant on population assumptions. However, errors in calibration would be systemic across all groups and would overall bias results toward the null. The finding of a graded relationship between kidney function category and clinical outcomes that is consistent with clinical expectations supports the use of these calibration factors. An additional weakness is that estimating equations by definition rely on assumptions regarding the relationship between demographic characteristics and muscle mass to determine GFR [23]; however, confirmation of results using the newly developed CKD-EPI GFR estimating equation is reassuring. Further weaknesses include a substantial portion of baseline participants without serum creatinine assessment at the time of their follow-up visit and the absence of data on albuminuria. Additionally there are multiple reasons for creatinine change over time, including both progression and remission of kidney disease, measurement error, use of medications affecting creatinine secretion, altered creatinine generation due to body composition changes, and major alterations in dietary intake. We are unable to differentiate among these. Finally, we utilize three-year follow-up rather than reassessment at three months as suggested in clinical guidelines as more frequent measures are unavailable.

This study also has several notable strengths. We utilize a well-characterized cohort with generalizability to the overall US population for the analyses. There is thorough event ascertainment and extensive data on traditional cardiovascular disease risk factors. Finally, participants were considered to be medically stable and generally healthy at the time of enrollment, adding to generalizability.

## Conclusion

In conclusion, the major findings in this manuscript are: 1) There is moderate change in classification of CKD when using two rather than one eGFR assessment in this longitudinal cohort, with 23.8% of individuals with baseline eGFR below 60 mL/min per 1.73m^2 ^who were able to attend a follow-up 3 years later having eGFR ≥60 mL/min per 1.73m^2 ^at that time; and 2) Individuals with persistent reduction in eGFR, even those with only mildly reduced eGFR, are at increased risk of cardiovascular outcomes and mortality, while individuals with an eGFR < 60 mL/min per 1.73m^2 ^at any time are at intermediate risk of cardiovascular events and mortality.

These data support the use of most recent eGFR as well as longitudinal changes in eGFR to assign risk, while classifying individuals with any eGFR result < 60 mL/min per 1.73m^2 ^at intermediate risk.

## Competing interests

The authors declare that they have no competing interests.

## Authors' contributions

DEW and MJS conceived of and designed the study. DEW, HT and MJS analyzed and interpreted the data. DEW and MK drafted the manuscript. DNS, ASL and MJS revised the manuscript for important intellectual content. All authors read and approved the final manuscript.

## Pre-publication history

The pre-publication history for this paper can be accessed here:

http://www.biomedcentral.com/1471-2369/10/26/prepub
